# Chronic hepatitis B virus infection and risk of chronic kidney disease: a population-based prospective cohort study of 0.5 million Chinese adults

**DOI:** 10.1186/s12916-018-1084-9

**Published:** 2018-06-18

**Authors:** Jiahui Si, Canqing Yu, Yu Guo, Zheng Bian, Chenxi Qin, Ling Yang, Yiping Chen, Li Yin, Hui Li, Jian Lan, Junshi Chen, Zhengming Chen, Jun Lv, Liming Li, on behalf of the China Kadoorie Biobank Collaborative Group

**Affiliations:** 1 Department of Epidemiology and Biostatistics, School of Public Health, Peking University Health Science Center, 38 Xueyuan Road, Beijing, 100191 China; 2 Chinese Academy of Medical Sciences, Beijing, China; 3 Clinical Trial Service Unit & Epidemiological Studies Unit (CTSU), Nuffield Department of Population Health, University of Oxford, Oxford, UK; 4 Hunan Center for Disease Control & Prevention, Changsha, Hunan China; 5 Liuzhou Traditional Chinese Medical Hospital, Liuzhou, Guangxi China; 6 Liuzhou Center for Disease Control & Prevention, Liuzhou, Guangxi China; 7 China National Center for Food Safety Risk Assessment, Beijing, China; 8 Peking University Institute of Environmental Medicine, Beijing, China

**Keywords:** Chronic hepatitis B virus infection, Chronic kidney disease, Prospective cohort study

## Abstract

**Background:**

Existing evidence remains inconclusive as to the association between chronic hepatitis B virus (HBV) infection and the risk of chronic kidney disease (CKD). We prospectively examined the association between chronic HBV infection and CKD risk, and the joint associations of HBV infection with established risk factors of several lifestyle factors and prevalent diseases on CKD risk.

**Methods:**

Participants from the China Kadoorie Biobank were enrolled during 2004–2008 and followed up until 31 December 2015. After excluding participants with previously diagnosed CKD, cancer, heart disease, and stroke at baseline, the present study included 469,459 participants. Hepatitis B surface antigen (HBsAg) was qualitatively tested at baseline. Incident CKD cases were identified mainly through the health insurance system and disease and death registries.

**Results:**

During a median follow-up of 9.1 years (4.2 million person-years), we documented 4555 incident cases of CKD. Cox regression yielded multivariable-adjusted hazard ratios (HRs) and 95% confidence intervals (CIs). Compared with HBsAg-negative participants, the multivariable-adjusted HR (95% CI) for CKD was 1.37 (1.18, 1.60) for HBsAg-positive participants. The association was stronger in men (HR = 1.77; 95% CI: 1.43, 2.20) than in women (HR = 1.10; 95% CI: 0.88, 1.36). HBsAg-positive participants, with or without hepatitis or cirrhosis, whether or not under treatment, all showed increased risk of developing CKD. We observed positive additive interactions of HBsAg positivity with smoking, physical inactivity, or diabetes on CKD risk. Compared with HBsAg-negative participants who were nonsmokers, more physically active, or did not have diabetes at baseline, the greatest CKD risk for HBsAg-positive participants was for those who were smokers (HR = 1.85; 95% CI: 1.44, 2.38), physically inactive (HR = 1.91; 95% CI: 1.52, 2.40), or diabetic (HR = 6.11; 95% CI: 4.47, 8.36).

**Conclusions:**

In countries with a high endemicity of HBV infection, kidney damage associated with chronic HBV infection should be a non-negligible concern. Our findings also highlight the importance of health advice on quitting smoking, increasing physical activity, improving glucose control, and early screening for CKD in people with chronic HBV infection.

**Electronic supplementary material:**

The online version of this article (10.1186/s12916-018-1084-9) contains supplementary material, which is available to authorized users.

## Background

Chronic kidney disease (CKD), which has important health and economic implications [1], had a prevalence of 10.8% in China in 2010 [2], as high as that in developed countries such as the USA (13.1%) and Norway (10.2%) [3, 4]. CKD is a general term for heterogeneous disorders and defined based on the presence of kidney damage or decreased kidney function for 3 months or more [5]. Several risk factors for CKD have been identified, including diabetes, hypertension, older age, tobacco smoking, obesity, cardiovascular diseases, and nephrotoxic drugs or toxins [5, 6]. However, possible effects of more adverse factors on the CKD risk remain to be clarified.

Hepatitis B virus (HBV) infection has been shown to have negative impacts on renal funtion [7–9]. A recently published systematic review showed that two prospective studies consistently linked HBV infection to increased risk of end-stage renal disease [10]. However, another two prospective studies examining the association between HBV infection and a broader CKD outcome showed mixed results [11, 12].

China is a higher-intermediate HBV endemicity country, with an estimated seroprevalence of hepatitis B surface antigen (HBsAg) of 5.49% [13]. If chronic HBV infection is causally related to CKD risk, this may partially explain the high burden of CKD and may guide prevention efforts in China. In the present study, we prospectively examined the association between chronic HBV infection and CKD risk in the China Kadoorie Biobank (CKB) study of 0.5 million adults. We additionally examined the joint associations of HBV infection with established risk factors of several lifestyle factors and prevalent diseases on CKD risk, applying both multiplicative and additive interaction analyses.

## Methods

### Study population

Detailed descriptions of the CKB have been given elsewhere [14, 15]. Briefly, a total of 512,891 participants aged 30–79 years were recruited in 2004–2008 from five urban and five rural regions in China. Eligible participants were invited to a community assessment center and completed an interviewer-administered electronic questionnaire, physical measurements, and blood spot tests for random blood glucose and HBsAg. A 10 ml non-fasting blood sample for each participant was also collected and shipped to the central blood repository for long-term storage. The study protocol was approved by the Ethics Review Committee of the Chinese Center for Disease Control and Prevention (Beijing, China) and the Oxford Tropical Research Ethics Committee, University of Oxford (UK). All participants provided written informed consent before taking part in the study.

In the present analysis, we excluded participants who reported having been diagnosed with CKD (*n* = 7577), heart disease (*n* = 15,472), stroke (*n* = 8884), or cancer (*n* = 2577) by a qualified doctor. We also excluded participants with missing data or an unclear result for HBsAg (*n* = 11,136) or missing data for body mass index (BMI; *n* = 2), plus one participant who was lost to follow-up shortly after baseline (*n* = 1). After these exclusions, 469,459 participants remained for the final analyses.

### Assessment of exposure

At baseline, the whole venous blood of participants was qualitatively tested for HBsAg using on-site rapid test strips (ACON dipstick, USA) (negative, positive, or unclear). All participants also completed interviewer-administered laptop-based questionnaires and were asked if they had ever been diagnosed with chronic hepatitis (not limited to chronic viral hepatitis) or liver cirrhosis (yes or no) by a doctor. For those who reported a prior medical history of the disease, we further asked their age at the first diagnosis, and if they were still on treatment (not specifically anti-HBV treatment; yes or no).

### Assessment of covariates

Covariate information was obtained from the baseline questionnaire, including socio-demographic status (age, sex, education, occupation, household income, and marital status), lifestyle behaviors (alcohol consumption, tobacco smoking, physical activity, and intake of red meat, fresh fruit, and fresh vegetables), personal medical history (hypertension, diabetes, and rheumatic arthritis) and women’s menopause status. In the present analysis, we included in the current smoker category former smokers who had stopped smoking due to illness to avoid a misleadingly elevated risk. The daily level of physical activity was calculated by multiplying the metabolic equivalent tasks (METs) value for a particular type of physical activity by hours spent on that activity per day and summing the MET-hours for all activities.

Trained staff undertook baseline measurements of body weight, height, waist circumference, and blood pressure. BMI was calculated as weight in kilograms divided by the square of the height in meters. Prevalent hypertension was defined as measured systolic blood pressure ≥140 mmHg, measured diastolic blood pressure ≥90 mmHg, self-reported prior diagnosis of hypertension, or self-reported use of antihypertensive medication at baseline. Prevalent diabetes was defined as measured fasting blood glucose ≥7.0 mmol/l, measured random blood glucose ≥11.1 mmol/l, or self-reported prior diagnosis of diabetes at baseline.

### Ascertainment of incident chronic kidney disease

Incident CKD cases were identified by linking local disease and death registries, linking the national health insurance (HI) system, and by active follow-up (i.e., visiting local communities or directly contacting participants). In particular, the electronic linkage with the HI claims database is one of the most important means of ascertaining CKD cases in the present study. The HI data are comprehensive and contain information on all diagnoses and treatments prescribed to patients who sought health care in a hospital. Successful linkage to the HI system was achieved for more than 96% of the CKB participants in 2015, which was similar across ten survey sites. Trained staff blinded to baseline information coded all diagnoses using the International Classification of Diseases, Tenth Revision (ICD-10). The outcome of the present analysis was CKD, including diabetes mellitus with renal complications (E10.2, E11.2, E12.2, E13.2, and E14.2), hypertensive renal disease (I12 and I13), glomerular disease (N03, N04, N05, and N07), renal tubulo-interstitial disease (N11, N12, N13, N14, and N15), and renal failure (N18 and N19).

### Statistical analysis

Participants contributed person-time from baseline until the date of CKD diagnosis, death, loss to follow-up, or 31 December 2015, whichever came first. We used a multivariable Cox proportional hazards model to estimate the hazard ratio (HR) and 95% confidence interval (CI), with age as the underlying time scale, stratified by 5-year age groups and ten survey sites. The proportional hazards assumption for the Cox model was checked using Schoenfeld residuals, and no violation was found.

Multivariable models for association between chronic HBV infection and CKD risk were adjusted for age; sex (for whole cohort); level of education; marital status; alcohol consumption; smoking status; physical activity; frequencies of intake of red meat, fresh fruit, and fresh vegetables; menopausal status (for women only); BMI; prevalent diabetes; and prevalent hypertension. We further explored whether the association was mediated by the presence of chronic hepatitis or cirrhosis by adjusting for a composite variable of disease duration and treatment status at baseline.

In the sensitivity analyses, we excluded participants whose outcome occurred during the first 2 years of follow-up. We additionally adjusted for occupation, household income, waist circumference, systolic blood pressure, and prevalent rheumatic arthritis at baseline separately. We further included participants with medical histories of heart disease or stroke at baseline and additionally adjusted for these two variables. The risk estimates did not change materially (data not shown).

We examined whether the association of HBsAg status with CKD risk differed by demographics, lifestyles, and co-morbidities on both multiplicative and additive scales. Prespecified baseline subgroups included sex (men or women), age at baseline (<50 or ≥50 years), residence (urban or rural), smoking status (daily smoker or not), alcohol consumption (daily drinker or not), level of physical activity (categorized using sex-specific tertile cutoffs, the lowest tertile or others), BMI (<24.0 or ≥24.0 kg/m^2^), prevalent diabetes (presence or absence), and prevalent hypertension (presence or absence). We tested multiplicative interaction by using likelihood ratio test comparing models with and without a cross-product term. We assessed additive interaction by estimating the relative excess risk due to interaction (RERI) [16]. A RERI of 0 indicates no interaction on the additive scale and >0 indicates a synergistic interaction. We further decomposed the joint effect of two exposures, that is, comparing both exposures present to both absent, into three components: the proportions attributable to HBsAg positivity alone, to the prespecified baseline variable alone, and to their interaction [16, 17].

We used Stata version 14.2 (StataCorp, TX, USA) to analyze the data. Statistical significance was set at two-tailed *P* < 0.05.

## Results

Of all 469,459 participants, 41.0% were men, and 56.4% resided in rural areas. HBsAg-positive participants were more likely to be men and urban residents, and report having prevalent chronic hepatitis or liver cirrhosis. Among participants with chronic hepatitis or liver cirrhosis, HBsAg-positive participants were more likely to be under treatment at baseline (Table 1).

**Table 1:** Baseline characteristics according to HBsAg status at baseline for 469,459 participants

	HBsAg negative	HBsAg positive	*p* value
Number of participants	454,588	14,871	
Age (years)	51.1	48.6	<0.001
Men (%)	40.8	46.9	<0.001
Rural area (%)	56.7	48.7	<0.001
Married (%)	90.9	90.3	0.024
Middle school and higher (%)	49.5	47.4	<0.001
Daily smoker (%)			
Men	67.7	68.4	0.231
Women	2.6	2.9	0.104
Daily drinker (%)			
Men	20.9	20.2	0.165
Women	0.9	1.1	0.046
Physical activity (MET-h/day)	21.5	21.4	0.650
Weekly consumption^*^			
Red meat (day)	3.7	3.7	0.002
Fresh vegetables (day)	6.8	6.8	0.738
Fresh fruit (day)	2.5	2.5	0.014
Body mass index (kg/m^2^)^†^	23.6	23.5	<0.001
Postmenopausal (%)	50.7	50.9	0.521
Prevalent diabetes (%)^†^	5.4	5.7	0.075
Prevalent hypertension (%)^†^	33.9	32.3	< 0.001
Prevalent rheumatic arthritis (%)	1.9	1.8	0.448
Prevalent chronic hepatitis or cirrhosis (%)	0.8	10.8	< 0.001
Medical history (years)^‡^	17.2	12.6	< 0.001
Under treatment at baseline (%)^‡^	10.4	19.1	< 0.001

All variables were adjusted for age, sex, and survey sites, as appropriate

*HBsAg* hepatitis virus B surface antigen, *MET* metabolic equivalent of task

^*^Weekly consumption of red meat, fresh vegetables, and fresh fruit was calculated by assigning participants to the midpoint of their consumption category

^†^Variables obtained or partly obtained by physical measurements. Other variables were obtained through the questionnaire

^‡^Among participants with prevalent chronic hepatitis or cirrhosis at baseline

During a median of 9.1 years (interquartile range 1.88 years; 4.2 million person-years) of follow-up, we documented 1762 incident CKD cases among men and 2793 among women. In the multivariable-adjusted model, HBsAg positivity was significantly associated with a higher risk of incident CKD (Table 2). In all eligible participants, compared with HBsAg-negative participants, the HRs (95% CIs) for CKD were 1.37 (1.18, 1.60) for HBsAg-positive participants. The association between HBsAg status and CKD risk was stronger in men (HR = 1.77; 95% CI: 1.43, 2.20) than in women (HR = 1.10; 95% CI: 0.88, 1.36) (*P* = 0.007 for interaction with sex). Further adjustment for the presence of chronic hepatitis or cirrhosis at baseline did not change the association materially.

**Table 2:** HRs (95% CIs) for incident chronic kidney disease according to HBsAg status among 469,459 participants

	HBsAg negative	HBsAg positive	*p* value
Whole cohort			
Number of cases	4381	174	
Cases/PY (1000)	1.07	1.33	
Age and sex adjusted	1.00	1.36 (1.17, 1.59)	<0.001
Multivariable adjusted^*^	1.00	1.37 (1.18, 1.60)	<0.001
+ Presence of hepatitis or cirrhosis^†^	1.00	1.33 (1.14, 1.55)	<0.001
Men			
Number of cases	1671	91	
Cases/PY (1000)	1.02	1.54	
Age adjusted	1.00	1.81 (1.46, 2.24)	<0.001
Multivariable adjusted^*^	1.00	1.77 (1.43, 2.20)	<0.001
+ Presence of hepatitis or cirrhosis^†^	1.00	1.72 (1.38, 2.14)	<0.001
Women			
Number of cases	2710	83	
Cases/PY (1000)	1.11	1.15	
Age adjusted	1.00	1.08 (0.86, 1.34)	0.517
Multivariable adjusted^*^	1.00	1.10 (0.88, 1.36)	0.414
+ Presence of hepatitis or cirrhosis^†^	1.00	1.06 (0.85, 1.33)	0.606

*CI* confidence interval, *HBsAg* hepatitis virus B surface antigen, *HR* hazard ratio, *PY* person-year

^*^Multivariable model was adjusted for: age (years); sex (men or women, for whole cohort); level of education (no formal school, primary school, middle school, high school, college, or university or higher); marital status (married, widowed, divorced or separated, or never married); alcohol consumption (less than weekly drinker, weekly drinker, daily drinker with an intake of <15, 15–29, 30–59, or ≥60 g/day); smoking status (nonsmoker, former smoker having quit smoking ≥5 or <5 years previously, or current daily smoker smoking <15, 15–24, or ≥25 cigarettes or equivalents per day); physical activity (MET-h/day); intake frequencies of red meat, fresh fruit, and fresh vegetables (daily, 4–6 days/week, 1–3 days/week, monthly, or rarely or never); body mass index (kg/m^2^); menopausal status (premenopausal, perimenopausal, or postmenopausal; for women only); prevalent diabetes; and prevalent hypertension at baseline (presence or absence)

^†^A composite variable of disease status (absence or presence), duration (< 15 or ≥ 15 years), and treatment status at baseline (no or yes)

We further examined the joint association of HBsAg status and presence of chronic hepatitis or cirrhosis and its treatment status with CKD risk (Table 3). Compared with HBsAg-negative participants without chronic hepatitis or cirrhosis, those who were HBsAg positive and had chronic hepatitis or cirrhosis under treatment at baseline had the greatest risk of developing CKD (HR = 4.74; 95% CI: 2.68, 8.36). The magnitude of risk was similar in men and women (*P* = 0.061 for interaction with sex). Male participants who were HBsAg positive but without chronic hepatitis or cirrhosis or not on treatment also showed a moderate increase in the CKD risk. HBsAg-positive participants who had been diagnosed with chronic hepatitis or cirrhosis for ≥15 years had a slightly higher risk of developing CKD compared with those diagnosed for <15 years (see Additional file 1).

**Table 3:** HRs (95% CIs) for incident chronic kidney disease according to HBsAg status and presence of chronic hepatitis or cirrhosis and its treatment status at baseline for 469,459 participants

	HBsAg negative		HBsAg positive		
	Without hepatitis or cirrhosis	With hepatitis or cirrhosis	Without hepatitis or cirrhosis	With hepatitis or cirrhosis	
				Without treatment	With treatment
Whole cohort					
Number of cases	4339	42	143	19	12
Cases/PY (1000)	1.07	1.28	1.22	1.62	5.03
HR (95% CI)	1.00	0.99 (0.73, 1.35)	1.27 (1.08, 1.51)	1.57 (1.00, 2.46)	4.74 (2.68, 8.36)
*p* value	–	0.961	0.004	0.052	<0.001
Men					
Number of cases	1652	19	72	12	7
Cases/PY (1000)	1.02	0.99	1.41	1.86	4.26
HR (95% CI)	1.00	0.85 (0.54, 1.34)	1.63 (1.29, 2.07)	2.10 (1.19, 3.72)	4.64 (2.20, 9.80)
*p* value	–	0.484	<0.001	0.011	<0.001
Women					
Number of cases	2687	23	71	7	5
Cases/PY (1000)	1.11	1.69	1.08	1.32	6.71
HR (95% CI)	1.00	1.20 (0.79, 1.81)	1.03 (0.82, 1.31)	1.15 (0.55, 2.42)	5.25 (2.18, 12.66)
*p* value	–	0.390	0.775	0.712	<0.001

*CI* confidence interval, *HBsAg* hepatitis virus B surface antigen, *HR* hazard ratio, *PY* person-year

Multivariable model was adjusted for: age (years); sex (men or women, for whole cohort); level of education (no formal school, primary school, middle school, high school, college, or university or higher); marital status (married, widowed, divorced or separated, or never married); alcohol consumption (less than weekly drinker, weekly drinker, daily drinker with an intake of <15, 15–29, 30–59, or ≥60 g/day); smoking status (nonsmoker, former smoker having quit smoking ≥5 or <5 years previously, or current daily smoker smoking <15, 15–24, or ≥25 cigarettes or equivalents per day); physical activity (MET-h/day); intake frequencies of red meat, fresh fruit, and fresh vegetables (daily, 4–6 days/week, 1–3 days/week, monthly, rarely, or never); body mass index (kg/m^2^); menopausal status (premenopausal, perimenopausal, or postmenopausal; for women only); prevalent diabetes; and prevalent hypertension at baseline (presence or absence)

We examined the association between HBsAg status and CKD risk according to potential baseline risk factors. Notably, the effect of HBsAg positivity interacted with the effect of smoking, physical inactivity, or prevalent diabetes on CKD risk, on multiplicative and/or additive scales (Table 4). The positive association between HBsAg status and CKD risk was stronger in those who were smokers, less physically active, or diabetic. Compared with HBsAg-negative participants who were nonsmokers, more physically active, or did not have diabetes at baseline, HBsAg-positive participants had the greatest risk of developing CKD if they were smokers (HR = 1.85; 95% CI: 1.44, 2.38), physically inactive (HR = 1.91; 95% CI: 1.52, 2.40), or diabetic (HR = 6.11; 95% CI: 4.47, 8.36). We observed positive additive interactions of HBsAg positivity with smoking, physical inactivity, or diabetes on CKD risk, with all corresponding RERI > 0. The proportion of risk (%) in the doubly exposed group attributable to the interaction with HBsAg positivity was 66.3 (32.4, 100.3) for smoking, 79.1 (50.5, 107.7) for physical inactivity, and 48.0 (27.7, 68.3) for diabetes, respectively. No statistically significant interaction was observed for the following baseline factors on both multiplicative and additive scales: age, rural or urban residence, alcohol consumption, BMI, and prevalent hypertension (see Additional file 2).

**Table 4:** HRs (95% CIs) for chronic kidney disease in relation to HBsAg status by potential baseline risk factors among 469,459 participants

Variable of interest	HBsAg	Number of cases	Cases/PY (1000)	Multiplicative interaction				Additive interaction			
				Stratum-specific HR (95% CI)	*p*_Int-M_^*^	HR (95% CI)	*p*_Int-A_^†^	RERI	Attributable proportion, %		
									HBV infection	Variable of interest	Additive interaction
Daily smoking											
No	Negative	3166	1.09	1.00	0.030	1.00	0.030	0.56 (0.05, 1.07)	25.5 (−5.0, 56.1)	8.2 (− 16.3, 32.6)	66.3 (32.4, 100.3)
No	Positive	108	1.20	1.20 (0.99, 1.45)		1.22 (1.00, 1.47)					
Yes	Negative	1215	1.03	1.00		1.07 (0.97, 1.18)					
Yes	Positive	66	1.59	1.83 (1.42, 2.34)		1.85 (1.44, 2.38)					
Physical inactivity^‡^											
No	Negative	2721	0.99	1.00	0.003	1.00	0.004	0.72 (0.23, 1.21)	15.9 (−10.6, 42.3)	5.0 (−18.4, 28.4)	79.1 (50.5, 107.7)
No	Positive	97	1.07	1.15 (0.94, 1.41)		1.14 (0.93, 1.40)					
Yes	Negative	1660	1.24	1.00		1.05 (0.97, 1.12)					
Yes	Positive	77	1.90	1.80 (1.43, 2.27)		1.91 (1.52, 2.40)					
Prevalent diabetes											
No	Negative	3519	0.91	1.00	0.068	1.00	0.012	2.45 (0.53, 4.38)	5.4 (0.7, 10.2)	46.6 (25.9, 67.2)	48.0 (27.7, 68.3)
No	Positive	134	1.07	1.28 (1.07, 1.52)		1.28 (1.08, 1.52)					
Yes	Negative	862	4.11	1.00		3.38 (3.13, 3.66)					
Yes	Positive	40	6.54	1.77 (1.28, 2.44)		6.11 (4.47, 8.36)					

*CI* confidence interval, *HBsAg* hepatitis virus B surface antigen, *HR* hazard ratio, *PY* person-year, *RERI* relative excess risk due to interaction

^*^p value for multiplicative interaction

^†^*p* value for additive interaction

^‡^Defined as <13.4 or <12.4 MET-h/day for men or women respectively

Multivariable model was adjusted for: age (years); sex (men or women, for whole cohort); level of education (no formal school, primary school, middle school, high school, college, or university or higher); marital status (married, widowed, divorced or separated, or never married); alcohol consumption (less than weekly drinker, weekly drinker, daily drinker with an intake of <15, 15–29, 30–59, or ≥60 g/day); intake frequencies of red meat, fresh fruit, and fresh vegetables (daily, 4–6 days/week, 1–3 days/week, monthly, rarely, or never); body mass index (kg/m^2^); menopausal status (premenopausal, perimenopausal, or postmenopausal; for women only); and prevalent hypertension at baseline (presence or absence). The variables of smoking status (nonsmoker, former smoker having quit smoking ≥5 or <5 years previously, or current daily smoker smoking <15, 15–24, or ≥25 cigarettes or equivalents per day), physical activity (MET-h/day), and prevalent diabetes were adjusted for in the multivariable model except for their own subgroup analysis

## Discussion

In this large prospective Chinese adult cohort, we found that chronic HBV infection was associated with a 37% increased risk of CKD. The association was stronger in men than in women. In addition, the joint effects of chronic HBV infection with smoking, physical inactivity, or prevalent diabetes on CKD risk were more than the addition of the risk associated with each of these factors.

To our knowledge, only two prospective studies have examined the association between HBV infection and CKD risk. In a study using claims data from the Taiwan National Health Insurance Research Database, 17,796 adults with untreated HBV infection and 71,184 matched controls were compared and showed an increased risk of CKD associated with chronic HBV infection (HR = 2.58; 95% CI: 1.95, 3.42) during a mean 6.5 years of follow-up [11]. However, in another study conducted for 4329 adults who received regular healthy check-ups in a Chinese hospital, occult HBV infection, defined as seropositive for antibodies to the HBV core antigen, was not associated with CKD in the subsequent 5 years (odds ratio = 1.12; 95% CI: 0.65, 1.95) [12]. In the present study, we associated chronic HBV infection, characterized by HBsAg positivity, with a higher risk of incident CKD over a period of close to 10 years. The risk estimates of CKD did not change materially after adjusting for chronic hepatitis or cirrhosis at baseline, suggesting that these conditions might not be the causal intermediates from HBV infection to CKD. The presence of chronic hepatitis or cirrhosis and receiving treatment at baseline might be an indicator of long-term active virus replication with a higher risk of causing damage, and was associated with the greatest CKD risk in the present population. Also, we cannot rule out the possibility that some medications for chronic liver diseases are nephrotoxic. However, in the present study, HBsAg-positive participants, without hepatitis or cirrhosis or not on treatment at baseline, still showed an increased risk of developing CKD.

The pathogenesis of HBV-associated nephropathy remains unclear, and several mechanisms have been implicated. Steatosis, a typical feature of chronic HBV infection, could induce lipid peroxidation and increase plasma inflammatory biomarkers, leading to endothelial dysfunction and renal injury [18]. It is also suggested that HBV carriers are more likely to have increased insulin resistance and a higher circulating level of transforming growth factor ß, contributing to the potentiation of apoptosis and renal fibrosis [19, 20]. It has been shown that men are more likely to develop steatosis [21] and have a lower HBV clearance than women [22]. It is consistent with our findings that the association between HBV infection and CKD is stronger in men than women.

Our in-depth analyses suggested important interactions of chronic HBV infection with tobacco smoking, physical inactivity, or diabetes on the CKD risk on both multiplicative and additive scales. The additive interaction is more relevant to public health measures than the multiplicative interaction. We found that about two-thirds of CKD cases among a population with chronic HBV infection who smoke tobacco would occur if both exposures were present but not if only one or the other was present. A similar additive interaction was found for HBV infection with physical inactivity or with diabetes on the CKD risk. These findings imply that the public health consequences of quitting smoking, increasing physical activity, and improving glucose control would be larger in participants with chronic HBV infection.

The synergistic effects of chronic HBV infection with individual lifestyle factors and conditions on CKD risk are biologically plausible, consistent with previous studies that the pathogenesis of HBV-associated nephropathy depends on interactions between viral, host, and environmental factors [23]. The adverse impact of chronic HBV infection on CKD might be exacerbated by smoking-induced renal atherosclerosis [24] and endothelial dysfunction [25], and attenuated by reduced oxidative stress and reduced inflammation with increasing physical activity [26]. Similarly, HBV infection and hyperglycemia-induced metabolic and hemodynamic pathways might be interwoven together in the pathogenesis of kidney injury [27, 28]. The mechanisms for their synergistic effects on CKD still warrant further elucidation.

To the best of our knowledge, the present study has been, by far, the largest prospective study examining the association between chronic HBV infection and CKD risk. For the first time, this study provides compelling evidence of the synergistic effects of chronic HBV infection with tobacco smoking, physical inactivity, or diabetes on CKD risk. Strengths of the study include its prospective design, large sample size, long follow-up period, the inclusion of a geographically spread Chinese population living in urban and rural areas, and careful adjustment for potential confounders.

This study acknowledges some limitations. We used an on-site HBsAg rapid test because it was feasible for a large-scale population study, but there was the possibility of misclassification. However, measurement errors in the prospective study may be nondifferential on subsequent disease status and may have attenuated our findings towards the null. Participants were excluded from the study based on self-reporting of a prediagnosed CKD at baseline. Underreporting of the subclinical or early stage of CKD might exist, leading to overestimation of CKD incidence during the follow-up. However, after excluding participants whose outcome occurred during the first 2 years of follow-up, the risk estimates remained largely unchanged. Also, the incident cases of CKD in this study were mainly identified using a linkage with the HI system. Some asymptomatic or mild cases might have been missed, resulting in nondifferential outcome misclassification and attenuation of the effect estimates. Lack of detailed information about viral load, antiviral treatment (especially the use of nephrotoxic drugs), or any biochemistry data indicating liver function precluded further analysis.

## Conclusions

In this large prospective study, we found that participants with chronic HBV infection had an increased risk of CKD. HBV infection exhibited noticeable synergistic effects with smoking, less physical activity, or diabetes on the CKD risk. In countries with an intermediate and high endemicity of HBV infection, kidney damage associated with chronic HBV infection should be a non-negligible concern. Our study also highlights the importance of health advice on quitting smoking, increasing physical activity, improving glucose control, and early screening for the CKD in people with chronic HBV infection. More studies are warranted to confirm the results of this study and clarify the underlying mechanism of the association.

## Additional files

Additional file 1: Table. HRs (95% CIs) for incident chronic kidney disease according to HBsAg status and presence of chronic hepatitis or cirrhosis and its duration for 469,459 participants. (DOCX 17 kb)

Additional file 2: Table. Association between HBsAg status and risk of chronic kidney disease by potential baseline risk factors for 469,459 participants. (DOCX 19 kb)

Additional file 3: Text. Members of the China Kadoorie Biobank collaborative group. (DOCX 17 kb)

## Abbreviations

BMI: Body mass index
CI: Confidence interval
CKB: China Kadoorie Biobank
CKD: Chronic kidney disease
HBsAg: Hepatitis B surface antigen
HBV: Hepatitis B virus
HI: Health insurance
HR: Hazard ratio
ICD-10: International Classification of Diseases, Tenth Revision
MET: Metabolic equivalent task
RERI: Relative excess risk due to interaction
