# Supplementary material for: Chronic hepatitis B virus infection and risk of chronic kidney disease: a population-based prospective cohort study of 0.5 million Chinese adults
Source: BMC Med. 2018 Jun 18;16:93. doi: 10.1186/s12916-018-1084-9 (PMC6004660; doi:10.1186/s12916-018-1084-9)
Supplement: Supplementary file 1 — Table. HRs (95% CIs) for incident chronic kidney disease according to HBsAg status and presence of chronic hepatitis or cirrhosis and its duration for 469,459 participants. (DOCX 17 kb) [file 12916_2018_1084_MOESM1_ESM.docx]

# Additional file 2 Table. HRs (95% CIs) for incident chronic kidney disease according to HBsAg status and presence of chronic hepatitis or cirrhosis and its duration among 469 459 participants

|  | **HBsAg negative** | |  | **HBsAg positive** | | | | |
| --- | --- | --- | --- | --- | --- | --- | --- | --- |
|  | **Without hepatitis or cirrhosis** | **With hepatitis**  **or cirrhosis** | | |  | **Without hepatitis**  **or cirrhosis** | **With hepatitis or cirrhosis** | |
|  |  |  |  |  |  |  | **Medical history**  **< 15 years** | **Medical history**  **≥ 15 years** |
| **Whole cohort** |  |  | | |  |  |  |  |
| No. of cases | 4 339 | 42 | | |  | 143 | 18 | 13 |
| Cases/ PYs (/1 000) | 1.07 | 1.28 | | |  | 1.22 | 1.90 | 2.79 |
| HR (95% CI) | 1.00 | 0.99 (0.73, 1.35) | | |  | 1.27 (1.08, 1.51) | 1.82 (1.15, 2.90) | 2.70 (1.57, 4.66) |
| *p* value | - | 0.960 | | |  | 0.004 | 0.011 | <0.001 |
| **Men** |  |  | | |  |  |  |  |
| No. of cases | 1 652 | 19 | | |  | 72 | 10 | 9 |
| Cases/ PYs (/1 000) | 1.02 | 0.99 | | |  | 1.41 | 1.82 | 3.46 |
| HR (95% CI) | 1.00 | 0.85 (0.54, 1.34) | | |  | 1.63 (1.29, 2.07) | 2.15 (1.15, 4.02) | 3.50 (1.81, 6.75) |
| *p* value | - | 0.486 | | |  | <0.001 | 0.016 | <0.001 |
| **Women** |  |  | | |  |  |  |  |
| No. of cases | 2 687 | 23 | | |  | 71 | 8 | 4 |
| Cases/ PYs (/1 000) | 1.11 | 1.69 | | |  | 1.08 | 2.01 | 1.95 |
| HR (95% CI) | 1.00 | 1.20 (0.79, 1.81) | | |  | 1.04 (0.82, 1.31) | 1.63 (0.81, 3.27) | 1.88 (0.70, 5.02) |
| *p* value | - | 0.390 | | |  | 0.775 | 0.169 | 0.207 |

HR denotes hazard ratio; CI, confidence interval; HBsAg, hepatitis virus B surface antigen; and PYs, person-years.

Multivariable model was adjusted for: age (years); sex (men or women, for whole cohort); level of education (no formal school, primary school, middle school, high school, college, or university or higher); marital status (married, widowed, divorced or separated, or never married); alcohol consumption (less than weekly drinker, weekly drinker, daily drinker with an intake of<15, 15–29, 30–59, or ≥60 g/day); smoking status (nonsmoker, former smoker having quit smoking ≥5 or <5 years previously, or current daily smoker smoking <15, 15–24, or ≥25 cigarettes or equivalents per day); physical activity (MET-h/day); intake frequencies of red meat, fresh fruit and vegetables (daily, 4–6 days/week, 1–3 days/week, monthly, or rarely or never); body mass index (kg/m^2^), menopausal status (premenopausal, perimenopausal, or postmenopausal; for women only), prevalent diabetes, and prevalent hypertension at baseline(presence or absence).
