# Supplementary material for: Chronic hepatitis B virus infection and risk of chronic kidney disease: a population-based prospective cohort study of 0.5 million Chinese adults
Source: BMC Med. 2018 Jun 18;16:93. doi: 10.1186/s12916-018-1084-9 (PMC6004660; doi:10.1186/s12916-018-1084-9)
Supplement: Supplementary file 2 — Table. Association between HBsAg status and risk of chronic kidney disease by potential baseline risk factors for 469,459 participants. (DOCX 19 kb) [file 12916_2018_1084_MOESM2_ESM.docx]

# Additional file 3 Table. Association between HBsAg status and risk of chronic kidney disease by potential baseline risk factors among 469,459 participants.

| **Subgroups** | **HBsAg negative** | | |  | **HBsAg positive** | | |  | *p***_Int-M_^*^** | *p***_Int-A_^†^** | **RERI** |
| --- | --- | --- | --- | --- | --- | --- | --- | --- | --- | --- | --- |
|  | **No. of cases** | **Cases/ PYs (/1,000)** | **HR** |  | **No. of cases** | **Cases/ PYs (/1,000)** | **HR (95%CI)** |  |  |  |  |
| **Age, years** |  |  |  |  |  |  |  |  | 0.227 | 0.204 | 0.26 (-0.14, 0.65) |
| <50 | 1 419 | 0.73 | 1.00 |  | 66 | 0.89 | 1.24 (0.96, 1.58) |  |  |  |  |
| ≥50 | 2 962 | 1.38 | 1.00 |  | 108 | 1.91 | 1.47 (1.21, 1.78) |  |  |  |  |
| **Residence** |  |  |  |  |  |  |  |  | 0.339 | 0.351 | 0.21 (-0.23, 0.66) |
| Rural | 2 837 | 1.22 | 1.00 |  | 105 | 1.61 | 1.31 (1.07, 1.59) |  |  |  |  |
| Urban | 1 544 | 0.88 | 1.00 |  | 69 | 1.04 | 1.50 (1.18, 1.92) |  |  |  |  |
| **Alcohol consumption** |  |  |  |  |  |  |  |  | 0.703 | 0.994 | -0.002 (-0.59, 0.58) |
| Not daily | 4 060 | 1.09 | 1.00 |  | 159 | 1.34 | 1.36 (1.16, 1.59) |  |  |  |  |
| Daily | 321 | 0.88 | 1.00 |  | 15 | 1.24 | 1.60 (0.95, 2.70) |  |  |  |  |
| **Body mass index, kg/m^2^** |  |  |  |  |  |  |  |  | 0.583 | 0.532 | 0.14 (-0.29, 0.57) |
| <24.0 | 2 344 | 1.01 | 1.00 |  | 95 | 1.24 | 1.31 (1.07, 1.61) |  |  |  |  |
| ≥24.0 | 2 037 | 1.15 | 1.00 |  | 79 | 1.46 | 1.45 (1.16, 1.82) |  |  |  |  |
| **Baseline hypertension** |  |  |  |  |  |  |  |  | 0.265 | 0.786 | -0.05 (-0.41, 0.31) |
| No | 2 009 | 0.74 | 1.00 |  | 82 | 0.88 | 1.28 (1.02, 1.59) |  |  |  |  |
| Yes | 2 372 | 1.74 | 1.00 |  | 92 | 2.42 | 1.47 (1.19, 1.81) |  |  |  |  |

HBsAg, hepatitis virus B surface antigen; PYs, person-years; HR, hazard ratio; CI, confidence interval; and RERI, relative excess risk due to interaction.

^*^*p* value for multiplicative interaction.

^†^*p* value for additive interaction.

Multivariable model was adjusted for: age (years); sex (men or women, for whole cohort); level of education (no formal school, primary school, middle school, high school, college, or university or higher); marital status (married, widowed, divorced or separated, or never married); alcohol consumption (less than weekly drinker, weekly drinker, daily drinker with an intake of<15, 15–29, 30–59, or ≥60 g/day); smoking status (nonsmoker, former smoker having quit smoking ≥5 or <5 years previously, or current daily smoker smoking <15, 15–24, or ≥25 cigarettes or equivalents per day); physical activity (MET-h/day); intake frequencies of red meat, fresh fruit and vegetables (daily, 4–6 days/week, 1–3 days/week, monthly, or rarely or never); body mass index (kg/m^2^), menopausal status (premenopausal, perimenopausal, or postmenopausal; for women only), prevalent diabetes, and prevalent hypertension at baseline(presence or absence). The variable was adjusted for in the multivariable model except for their own subgroup analysis.
